# First person – Mrudula Dileep and Anamika Sharma

**DOI:** 10.1242/dmm.052813

**Published:** 2026-01-21

## Abstract

First Person is a series of interviews with the first authors of a selection of papers published in Disease Models & Mechanisms, helping researchers promote themselves alongside their papers. Mrudula Dileep and Anamika Sharma are co-first authors on ‘ Synergism of IP_3_R and Parkin mutants identifies mitochondrial oxidative stress as an early feature of Parkinson's disease’, published in DMM. Mrudula conducted the research described in the article while a project associate in the lab of Professor Gaiti Hasan at National Centre for Biological Sciences, Bengaluru, India, and is now a PhD student in Jacek Kuźnicki's lab at International Institute of Molecular and Cellular Biology (IIMCB), Warsaw, Poland, investigating calcium-iron homeostasis in zebrafish models of Parkinson's disease. Anamika conducted the research described in the article while a PhD student in the lab of Professor Gaiti Hasan at National Centre for Biological Sciences, Bengaluru, India, and is now a postdoctoral associate in Professor Stuart Lipton's lab at The Scripps Research Institute, La Jolla, CA, USA, investigating how lifestyle and environmental factors modulate cellular and molecular functions in the brain to influence neurological health and disease.


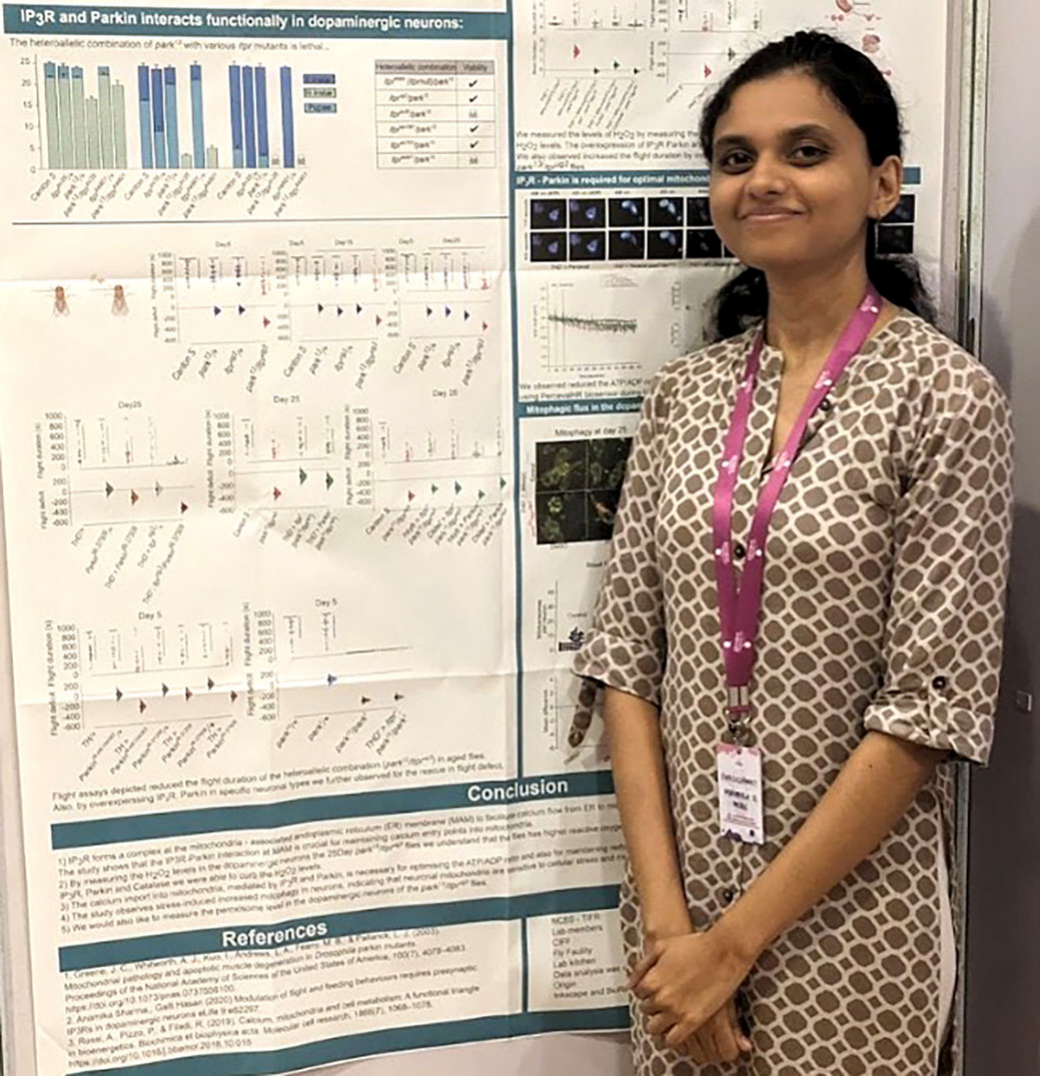


**Mrudula Dileep**

**Who or what inspired you to become a scientist?**

**M.D.:** It wasn't a childhood dream. I believe the plan was to always follow curiosity, and it led me to study science in high school, followed by biology, when I first learnt about the blue-print for creating life on earth – ‘the central dogma of molecular biology’. From here on, the questions never stopped … and I hope they don't.

**A.S.:** Biology classes in school seeded the curiosity of knowing fundamentals underlying cellular functions, and rapid advancements in technology to study cellular mechanisms watered the curiosity further.

**What is the main question or challenge in disease biology you are addressing in this paper? How did you go about investigating your question or challenge?**

We are determined to understand why the motor deficits were arising in the single-copy recessive mutants of IP_3_R and Parkin. At first, we decided to look into the functions of these proteins, which are calcium signalling and mitophagy, and, when they appeared normal, we realised that we might be looking into early stages of pathology development and shift our focus towards mitochondrial dysfunction and cellular stress.

**How would you explain the main findings of your paper to non-scientific family and friends?**

In diseases like Parkinson's, people lose the ability to control their movements properly. The tricky part is that by the time someone shows symptoms, they've already lost 60-70% of the brain cells that produce dopamine, a chemical messenger that helps control movement, among other things. We wanted to figure out what goes wrong before things get that bad. What are the earliest warning signs?

What we found is that the real trouble starts in the mitochondria, the tiny power plants inside cells. When these power plants get stressed, that's when problems begin. To test this, we increased levels of a protective enzyme called catalase in the dopamine-producing cells of fruit flies (our research model). This helped reduce the stress in their mitochondria, and, importantly, it reduced their movement problems too. So, our research suggests that catching and treating this mitochondrial stress early could help prevent or slow down movement issues in these kinds of diseases.

**What are the potential implications of these results for disease biology and the possible impact on patients?**

We've known for some time that in brain diseases like Parkinson's, harmful molecules called reactive oxygen species (which damage cells) play a major role in worsening the disease. Finding ways to fight this damage has been a key goal in research. What's encouraging is that we're seeing similar positive results across different studies. Our lab has observed the same kind of protective effect in another Parkinson's model using human stem cells, which suggests this approach might work more broadly (Musthafa et al., 2025). Now, to be clear, we still have a long way to go before we know if increasing catalase levels would be safe and effective in actual patients. But, based on what we're seeing in our research models, we think this approach of reducing cellular stress early on shows real promise for slowing down disease progression. If it translates to humans, it could potentially help patients maintain better movement control for longer.

… this approach of reducing cellular stress early on shows real promise for slowing down [Parkinson's] disease progression

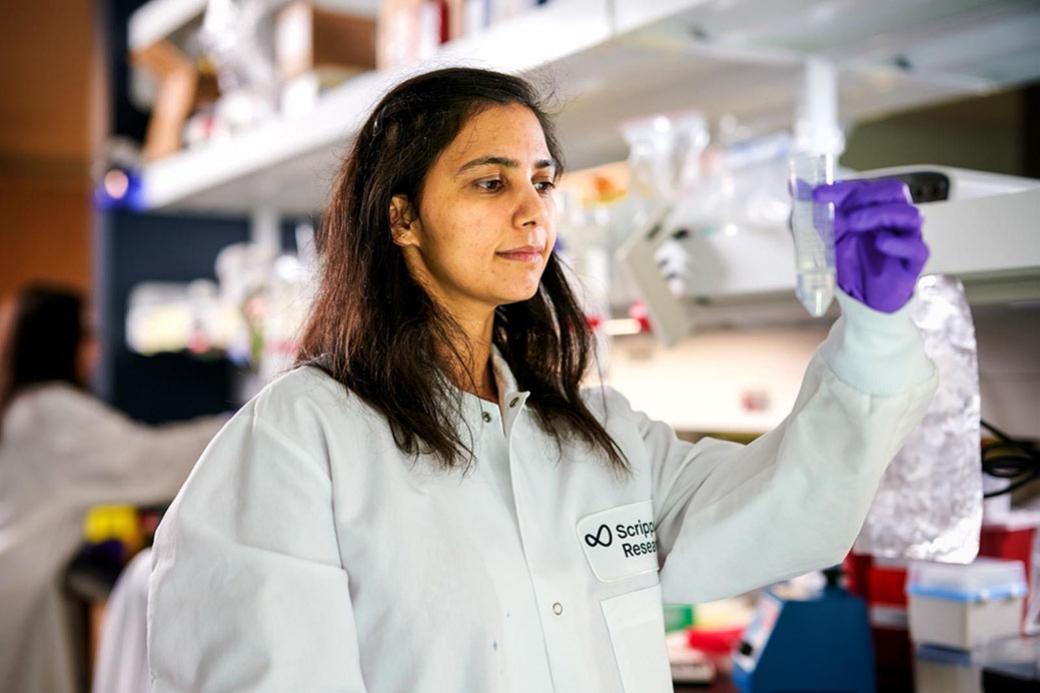


**Anamika Sharma**

**Figure:**
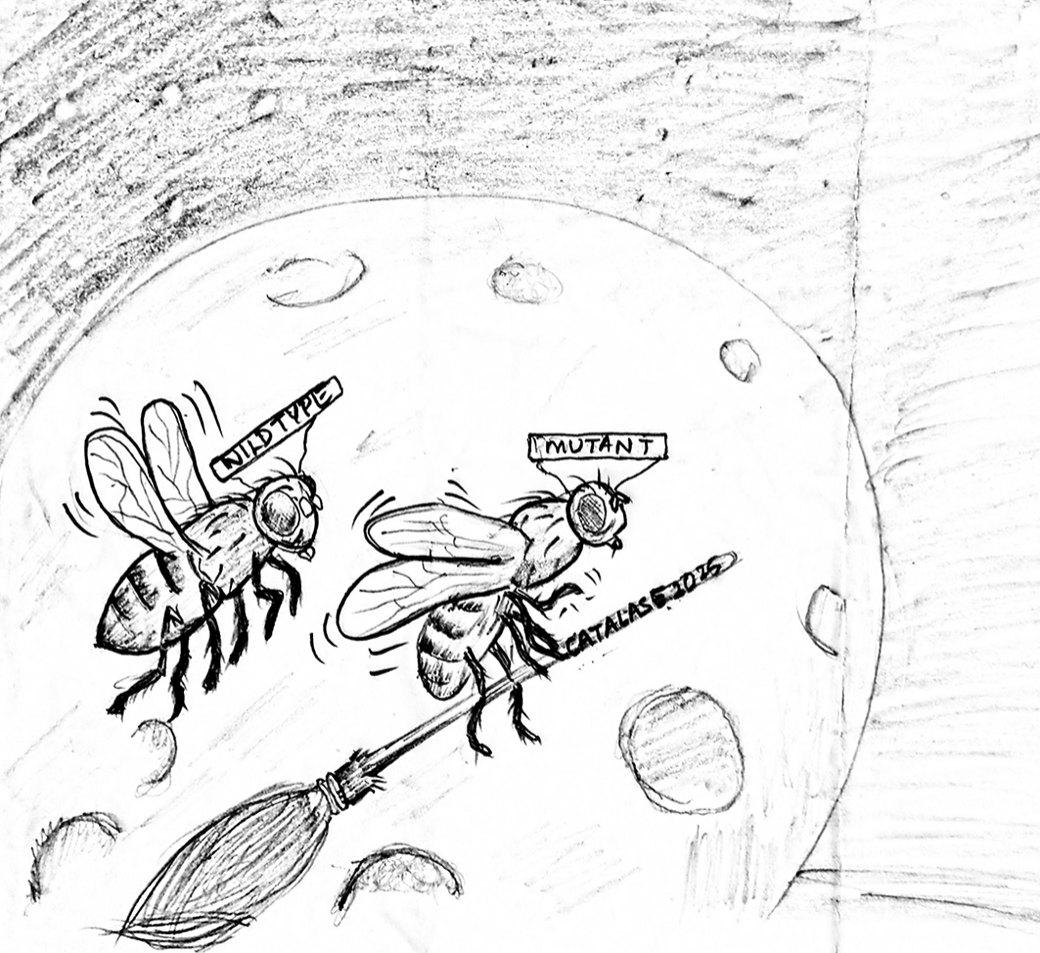
Fly me to the moon.

**Why did you choose DMM for your paper?**

Disease Models & Mechanisms felt like the perfect fit for our research. As the name suggests, the journal focuses on exactly what we're doing. We have a disease (Parkinson's), we're using a model (*Drosophila*) to study it, and we're exploring the underlying mechanisms (mitochondrial stress and early cellular changes). It seemed like a natural home for this work.

We're also really pleased that the paper has been accepted and published there. The whole process was exciting, and we feel the journal's focus aligns well with our approach to understanding disease progression at a fundamental level.

**Given your current role, what challenges do you face and what changes could improve the professional lives of other scientists in this role?**

**M.D.:** One of the biggest challenges we face is securing adequate funding for fundamental research. The current trends happening around the world with reduced funding is alarming. Often, basic science doesn't have the immediate, flashy applications that attract funding, but it's absolutely essential for understanding how diseases work at their core, which ultimately leads to better treatments down the line.

We also think more collaboration is essential across institutions and borders. Science works best when researchers can share resources, expertise and ideas freely. This is especially important for countries like India, where the scientific community is growing rapidly and producing excellent work, but sometimes lacks the infrastructure and funding that more established research hubs have. Better support for both early-career scientists and established researchers would make a huge difference. When scientists don't have to constantly worry about where their next grant is coming from, they can focus on asking bold questions and doing the kind of long-term, rigorous work that really advances our understanding of disease. Investing in fundamental science now pays dividends for decades to come.

**A.S.:** As an immigrant scientist working far from my family, ongoing uncertainty around visa status and research funding can be challenging and, at times, overwhelming. Greater stability in immigration pathways, along with increased funding opportunities and institutional support for early-career researchers, would go a long way toward improving the professional wellbeing and productivity of scientists in similar roles.

Investing in fundamental science now pays dividends for decades to come

**What's next for you?**

**M.D.:** I've started my PhD at IIMCB in Warsaw, Poland, where I'm continuing my work on Parkinson's disease and this time using zebrafish as my model organism. My research will focus on understanding how iron and calcium are regulated in Parkinson's mutants. It's exciting to build on what we learned from the fruit fly work and explore these questions in a different model system, which is a vertebrate that can give us new insights into the disease.

**A.S.:** I am currently a postdoctoral associate at Scripps, where I study how air pollution induces pathological redox-mediated changes in the brain that contribute to neurological disease. Moving forward, I plan to build on this work by securing independent funding through fellowship and career-development grants, with the goal of establishing my own research program focused on environmental drivers of brain health.

**Tell us something interesting about yourself that wouldn't be on your CV**

**M.D.:** I like to travel and hike.

**A.S.:** I grow my own veggies.
